# Spectroscopic Characteristics of Dissolved Organic Matter in Afforestation Forest Soil of Miyun District, Beijing

**DOI:** 10.1155/2016/1480857

**Published:** 2016-06-28

**Authors:** Shi-Jie Gao, Chen Zhao, Zong-Hai Shi, Jun Zhong, Jian-Guo Liu, Jun-Qing Li

**Affiliations:** ^1^College of Forestry, Beijing Forestry University, Beijing 100083, China; ^2^Key Laboratory of Urban Stormwater System, Beijing University of Civil Engineering and Architecture, Beijing 100044, China; ^3^Beijing Industrial Technician College, Beijing 100023, China

## Abstract

In this study, soil samples collected from different plain afforestation time (1 year, 4 years, 10 years, 15 years, and 20 years) in Miyun were characterized, including total organic carbon (TOC), total nitrogen (TN), total phosphorus (TP), available K (K^+^), microbial biomass carbon (MBC), and dissolved organic carbon (DOC). The DOM in the soil samples with different afforestation time was further characterized via DOC, UV-Visible spectroscopy, excitation-emission matrix (EEM) fluorescence spectroscopy, and ^1^H NMR spectroscopy. The results suggested that the texture of soil sample was sandy. The extracted DOM from soil consisted mainly of aliphatic chains and only a minor aromatic component. It can be included that afforestation can improve the soil quality to some extent, which can be partly reflected from the indexes like TOC, TN, TP, K^+^, MBC, and DOC. And the characterization of DOM implied that UV humic-like substances were the major fluorophores components in the DOM of the soil samples, which consisted of aliphatic chains and aromatic components with carbonyl, carboxyl, and hydroxyl groups.

## 1. Introduction

Urban forestry is often regarded as a key ecological asset of a city, which can significantly improve the ecoenvironment by lowering urban temperature by reducing heat island effect, improving air quality by absorbing pollutants, mitigating urban waterlogging by quick infiltration of stormwater runoff, and enhancing biodiversity by provision of habitat for living things. Each year, many efforts are put into urban afforestation in China, which led to the increase of the total forest area and the amount of forest reserves, further to make the cities more attractive and livable [1]. In case of Beijing, the capital of China, a large-scale plain reforestation project was launched, in which 133,000 hm^2^ afforestation forest was planned to increase each year from 2012 to 2015 [2].

Up to now, numerous studies have been carried out to investigate the characters of forest soil [3–5] and the dissolving organic matter in farmland soil [6], but few attentions were put to study the soil status of urban afforestation area. In order to assess the soil quality of urban afforestation, the dissolved organic matter (DOM), as the part of organic matter, was deemed to play a significant role in soil biological activity [7, 8]. And DOM can be regarded as a measure of soil quality and an integral part in a forest ecosystem, as its presence can increase the forest production by reducing erosion, increasing the elasticity, porosity, and water retention [9]. It is the most active and labile fraction with different molecular sizes, structures, and functional properties [8, 10] and exhibits heterogeneous nature as it is composed of the diverse and complicated compounds with different functional groups like aliphatic/phenolic hydroxy, amino, and carbohydrates [11, 12].

Generally, DOM plays an important role in the growth of plants due to its molecular structure. And it helps to break up clay and compacted soils, assists in transferring nutrients from the soil to the plant, and enhances water retention. Hence, to understand the dynamics of plant growth, it is necessary to know how DOM is distributed throughout the soil. In this paper, Miyun District, which was a typical afforestation area in Beijing, was chosen as sampling area. And the total organic carbon (TOC), total nitrogen (TN), total phosphorus (TP), available potassium (K^+^), microbial biomass carbon (MBC), and dissolved organic carbon (DOC) were determined. The DOM in the soil samples with different afforestation time was extracted and further characterized via dissolved organic carbon (DOC), UV-Visible spectroscopy, EEM fluorescence spectroscopy, and ^1^H NMR spectroscopy. Some valuable information on the soil quality of afforestation area can be provided, which will guide the future afforestation and corresponding management plan in Beijing.

## 2. Materials and Experimental

### 2.1. Sampling Sites Description

**Figure 1:**
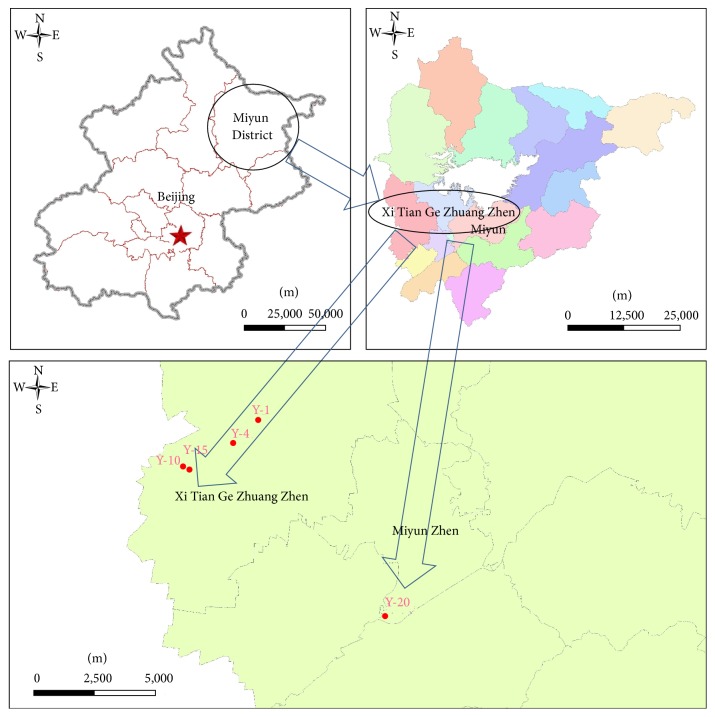
Schematic map of the selected area and the sampling sites.

As a typical afforestation area, Xi Tian Ge Zhuang town of Miyun District was selected as sampling region, as illustrated in Figure 1. The mean annual rainfall and average temperature of this area is 661.3 mm and 10.8°C, respectively. Five sampling regions with different plain afforestation time (1 year, 4 years, 10 years, 15 years, and 20 years) were selected in this study, and the samples were collected on June 27th, 2015 (Table 1). At each sampling area, 5 samples were collected with columns of 20 × 5 cm (long × diameter) via Soil Core Samplers (AMS samplers, American Falls, Idaho) [9, 13].

### 2.2. Pretreatment of the Soil Samples

In laboratory, after the visible roots, plant fragments, grass, and tones were removed, the soil samples were dried in the open air, passed through a 2 mm sieve, and stored at room temperature in airtight glassware containers [5].

### 2.3. The Determination of Some Soil Parameters

Soil bulk density was measured according to M.A. Rab using bulk density soil sampling kit (AMS samplers, American Falls, Idaho) [14]. The distribution of soil particle was analyzed with Mastersizer 3000 (Malvern Instrument Ltd., UK) to determine the content of clay, silt, and sand in the soil samples [15]. The pH values of the soil were measured in a suspension (water/dry soil, 2.5 : 1) using a PB-10 pH meter (Sartorius, Germany) [16]. Microbial biomass carbon (MBC) analysis was determined by the chloroform fumigation-extraction method [17]. Total organic carbon (TOC) was calculated by subtracting inorganic carbon (calculated from CaCO_3_ content) from total carbon by a Jena multi N/C 3100 analyzer [18]. The total N, P, and available P of the soil sample were measured using a AA3 continuous-flow analyzer (Seal Analytical Corporation, Germany). The available K was measured by flame atomic absorption spectrometric method via Perkin Elmer 9100 Atomic Absorption Spectrometry.

### 2.4. DOM Extraction and Analytical Methods

10.0 g soil sample was mixed with 50.0 mL distilled water in 100.0 mL centrifuge tube, which was shaken at 200 r/min in a reciprocal shaker for 16 h under room temperature (25°C), and then centrifuged at 12000 r/min for 20 min. The supernatant was moved out of centrifuge tube with a hydrophilic PVDF Millipore membrane filter (0.45 *μ*m) to carry out the following characterizations like dissolved organic carbon (DOC), UV-Visible spectroscopy, excitation-emission matrix (EEM) fluorescence spectroscopy, and even ^1^H NMR spectroscopy.

### 2.5. UV-Visible Spectroscopy

UV-Visible spectra of the DOM in filtered supernatant were recorded from 200 to 600 nm on a PerkinElmer Lambda 650S spectrophotometer using a 1.0 cm quartz cell [19]. The absorbance at 300 nm was adjusted to 0.02 to avoid inner filter effects [20]. Ultrapure water (Milli-Q, 18 MΩ·cm) was selected as the blank.

### 2.6. Excitation-Emission Matrix (EEM) Fluorescence Spectroscopy

The excitation and emission spectra of the DOM in filtered supernatant were measured on a Hitachi F-7000 fluorescence spectrophotometer using a 150 W xenon arc lamp as the light source. Both excitation and emission slits are 10 nm with a scan range from 200 nm to 550 nm. The scan rate was 1200 nm/min, and the photomultiplier tube voltage was 700 V. The standard quinine sulfate units (QSU) were introduced to determine the samples' relative fluorescent intensities; that is, 42.21 intensity unit is equivalent to one QSU (1 QSU = 1 *μ*g L^−1^ = 1 ppb quinine sulfate in 0.05 mol L^−1^ H_2_SO_4_) [21, 22]. The Rayleigh scatter effects were eliminated from the data set by adding zero to the EEMs in the two triangle regions (Em ≤ Ex + 20 nm and Em ≥ 2Ex − 10 nm) [23], along with Raman scatter that was avoided by subtracting the background value of the ultrapure water as blank [23, 24] to highlight the useful fluorescent information.

### 2.7. ^1^H NMR Spectroscopy

Agilent VacElut SPS 24 solid phase extraction (SPE) equipment with Bond Elut C-18 as sorbent was selected to isolate the DOM from the samples, in which 10.0 mL leaching liquor extracted from soil sample was pumped through the SPE column with the speed of 1.0 mL/min. Two 5.0 mL ultrapure water solvents were used to pass through the SPE column to wash off the residual salts. The DOM held in the C-18 packing of SPE column was eluted off with 6 mL solution matrix of water and methanol with volume ratio of 1 : 9. The reduced pressure was stained for 30 min to remove the residual solvent [21]. Finally, the extracted DOM was dried under N_2_ gas with Termovap Sample Concentrator (YGC-1217, Bao Jing Company) [22]. In order to conduct ^1^H NMR spectroscopy analysis, the obtained solid DOM extracts were dissolved in D_2_O (Jin Ouxiang Company) to avoid water peaks. Measurements of ^1^H NMR spectra of the DOM were performed on a Bruker 400 M NMR spectrometer. The pulse conditions of ^1^H NMR were listed as the following: operating frequency = 499.898 MHz, acquisition time = 2.045 s, recycle delay = 1.0 s, and line broadening = 1.0 Hz. The standard s2pul pulse sequence and baseline correction was applied. The functional groups in the ^1^H NMR spectra were identified on their corresponding chemical shifts (*δ*_H_) relative to that of the water (4.7 ppm) [25].

## 3. Results and Discussion

### 3.1. Soil Texture and Soil Quality Indicators

The basic physical and chemical properties of the soil samples are assessed. As listed in Table 2, it can be seen that all of the soil samples selected in this study are sandy texture, with a silt/sand ratio ranging from 73.89% to 92.64%, and the bulk densities were in the range of 1.45–1.57 g·cm^−3^, which matched well the previously reported values [26]. As shown in Table 3, the basic indexes of soil fertility, total organic carbon (TOC), total nitrogen (TN), total phosphorus (TP), and available potassium (K) are ranging from 2.64 to 4.52 g·kg^−1^, 0.03 to 0.05 g·kg^−1^, 0.05 to 0.48 g·kg^−1^, and 0.21 to 0.61 g·kg^−1^, respectively. The TOC increases with the increasing of the afforestation time when the afforestation time is less than ten year and then remains nearly constant when the time is up to ten year. The microbial biomass carbon analysis (MBC) and water extracted dissolved organic carbon (DOC) are in the range of 16.28 to 92.70 mg·kg^−1^ and 56.08 to 81.10 mg·kg^−1^, respectively. The highest MBC (92.70 mg·kg^−1^) is observed at sample Y-20. All of the basic physical and chemical properties of the samples are shown that the afforestation can improve biomass in soil.

### 3.2. Spectroscopic Characteristics of DOM in Soil Samples

#### 3.2.1. UV-Visible Spectroscopy

**Figure 2:**
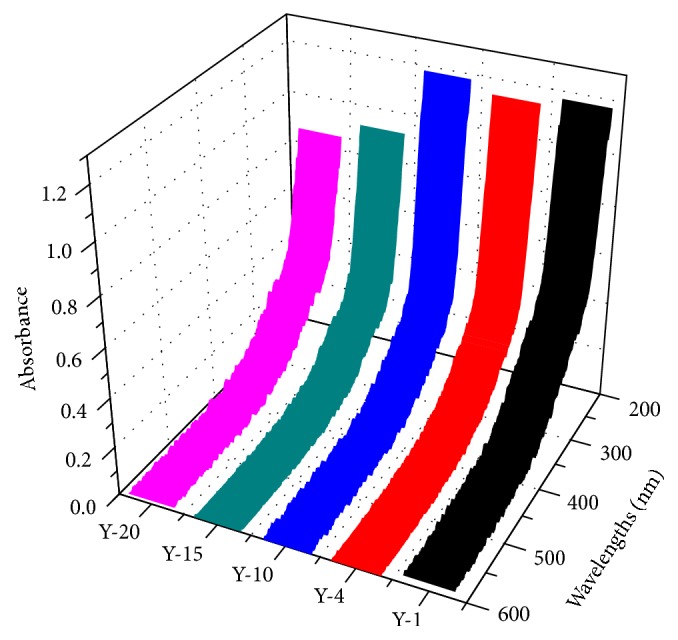
UV-Visible spectra of the soil samples.

As an efficient tool, the UV-Visible spectroscopy is employed to evaluate the composition and structure of DOM [27, 28]. As illustrated in Figure 2, all the UV-Visible spectrum absorbance of the five samples decreased with the wavelength, which can be found in other studies [5, 24, 29, 30]. Some small shoulder peaks can be found in the region of 250–300 nm, which might be contributed by phenolic, aromatic carboxylic, and polycyclic aromatic compounds (*π* → *π* ^*∗*^ transition) [31].

The spectral slope coefficients (*S*) calculated from the UV-Visible spectra data exhibited the light absorption efficiency of DOM as a function of the wavelength, which negatively relates to molecular weight of DOM [32, 33]. Jamieson et al. calculated *S* values within the spectra region of 275–295 nm to characterize the biochar-derived DOM isolated from soil [32], and Stedmon et al. calculated *S* values in the region from 300 to 650 nm to investigate the molecular weight of DOM in Danish coastal water bodies [34]. To avoid the use of spectral data near the detection limit of the instruments, the ratio of the slope of the shorter wavelength region (275–295 nm, *S*_275–295_) to that of the longer wavelength region (350–400 nm, *S*_350–400_) was calculated, and this dimensionless parameter can be called *S*_*R*_ [33].

The *S* and *S*_*R*_ values were calculated as follows:

(1) aλ=aλ0eSλ0−λ+K,aλ=2.303Aλl,SR=S275–295S350–400,

in which, *a*(*λ*) and *a*(*λ*_0_) represent absorption coefficients and absorption coefficients at reference wavelength, respectively. *λ* and *λ*_0_ are the reference wavelength (300 nm was selected in this study) and the selected wavelength (ranging from 240 nm to 400 nm), respectively. *A*(*λ*) represents the absorbance at wavelength *λ* (nm), *l* (m) is the optical path length (0.01 m in this study), and *K* is a background *d* parameter to improve the goodness of fitting.

The median of *S*_*R*_ values determined for the DOM samples of the five sits (Y-1, Y-4, Y-10, Y-15, and Y-20) was 0.64, 1.07, 0.92, 0.95, and 0.77, respectively, suggesting that the molecular weight of DOM in the soil samples is Y-1 > Y-20 > Y-10 > Y-15 > Y-4.

In order to explore the aromaticity of the DOM samples, SUVA_254_ was introduced as follows:

(2) $$\begin{array}{c} {SUVA}_{254}=\frac{a_{254}}{DOC}, \end{array}$$

where *a*_254_ (m^−1^) is the absorbance coefficient measured at 254 nm. The median values of SUVA_254_ were 5.39, 5.90, 4.83, 5.33, and 4.39 L mg C^−1^ m^−1^ for Y-1, Y-4, Y-10, Y-15, and Y-20, respectively. The previously reported SUVA_254_ values of wetland soil [35] and the agricultural soil [27] were 3.51–4.41 and 0.32–4.65, respectively, suggesting that the DOM in our study contained a greater amount of aromatic structures. And previous studies proposed that different landscapes were likely to produce different types of DOM [36, 37]; therefore, the SUVA_254_ values in this study could roughly imply that afforestation in suburban area can produce the aromatic substances. The highest values of SUVA_254_ were observed at Y-4, while the lowest values were observed at Y-20.

#### 3.2.2. Excitation-Emission Matrix (EEM) Fluorescence Spectroscopy

**Figure 3:**
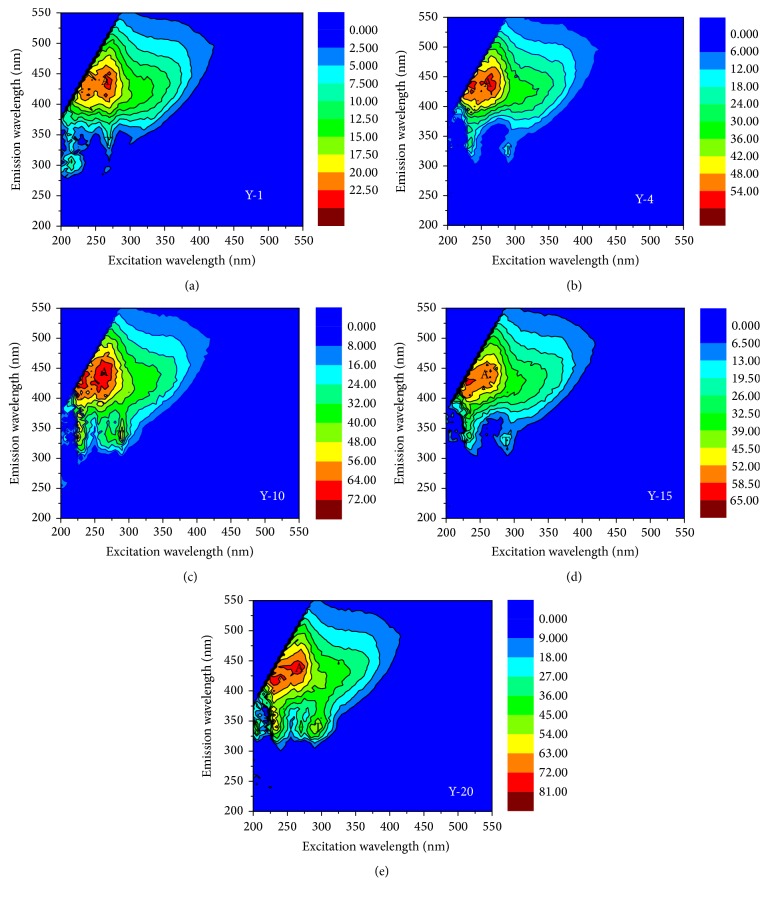
EEM fluorescence contour profiles of the five sites' DOM.

The excitation-emission matrix (EEM) fluorescence technique is powerful to provide sufficient information on DOM's molecular size, chemical composition, and aromaticity or aliphatic properties [5, 27]. EEM can identify humic-like (designated as A, C, and M) and protein-like fluorescence peaks (B and T), as listed in Table 4. EEM fluorescence spectra of the study areas samples were depicted in Figures 3(a)–3(e), suggesting that the UV humic-like substances were the primary fluorophores components in the DOM extracted from afforestation land samples [38]. The first identified peak (peak A) was located at Ex/Em of 257 nm/448 nm, 256 nm/440 nm, 262 nm/443 nm, 260 nm/425 nm, and 260 nm/435 nm for the Y-1, Y-4, Y-10, Y-15, and Y-20 samples, respectively, suggesting the existence of UV humic-like substances. The second typical peak (peak T) was observed at Ex/Em of 271 nm/347 nm, 280 nm/325 nm, 275 nm/338 nm, 279 nm/333 nm, and 271 nm/339 nm for the five samples stated above, respectively, implying the presence of tryptophan protein-like components [38]. Generally, DOM in soil is affected by some factors like local geology, land use, and microbial and human activities [38]. And it can be believed that the presence of tryptophan protein-like substances could further confirm the larger molecular weight DOM presented in the soil [39, 40].

Moreover, the fluorescence intensities of the five sites (Y-1, Y-4, Y-10, Y-15, and Y-20) for peak A were 24, 60, 78, 63, and 81 QSU, respectively, while the peak T fluorescence intensities of the five sites were 8.34, 91, 106, 76, and 124 QSU, respectively.

Three typical fluorescence indices like the fluorescence index (FI), the humification index (HIX), and the biological index (BIX) were selected to investigate the sources, the degree of maturation, and the influence from autochthonous biological activity of DOM, as listed in the following:

(3) FI=f450f500,HIX=HL,BIX=f380f430,

where *f*_450_ and *f*_500_ are the intensities at the emission wavelength of 450 nm and 500 nm at the excitation wavelength 370 nm, respectively [41, 42], while *f*_380_ and *f*_430_ are the fluorescence intensity at the emission wavelength of 380 nm and 430 nm at the excitation wavelength 310 nm, respectively [27, 38]. *H* and *L* represent the integral values from 435 to 480 nm and 300 to 345 nm at the excitation wavelength 254 nm, respectively [27, 43].

As listed in Table 5, the mean FI values of the five sites' soil samples were 1.54, 1.61, 1.46, 1.52, and 1.51 for Y-1, Y-4, Y-10, Y-15, and Y-20, respectively. Considering that the terrestrial and microbial end-member values were reported as 1.4 and 1.9 [41, 42], the FI values in this study suggested that the sources of DOM in the soil samples were possibly assigned to both terrestrial and microbial sources, which cannot be influenced by the afforestation time. The mean HIX values in this study were 14.70, 18.90, 2.17, 6.16, and 3.66 for Y-1, Y-4, Y-10, Y-15, and Y-20, respectively. It was believed that the high HIX values at the region of 10–16 are the indicator of the strongly humic organic substances (terrestrial origin), whereas low values (<4) imply the presence of autochthonous organic components [38–40].

Compared to the samples of the Y-10 and Y-20 sites, which mainly consisted of autochthonous organic matters (HIX values < 4.05), the DOM extracted from the soil samples of the other three sites (Y-1, Y-4, and Y-15) was composed of both allochthonous and autochthonous organic substances. Previous study reported that the allochthonous organic matters originated from incomplete decomposition of plant and animal residues, while the autochthonous organic ones may derive from the photosynthesis [40]. High BIX values (>1) correspond to autochthonous sources, while low BIX values (<1) imply low abundance of organic matter of biological origin [38–40]. The mean BIX values of the five sites' soil samples were 0.56, 0.48, 0.57, 0.43, and 0.67 for Y-1, Y-4, Y-10, Y-15, and Y-20, respectively, indicative of low abundance of biological origin organic components.

#### 3.2.3. ^1^H NMR Spectroscopy

**Figure 4:**
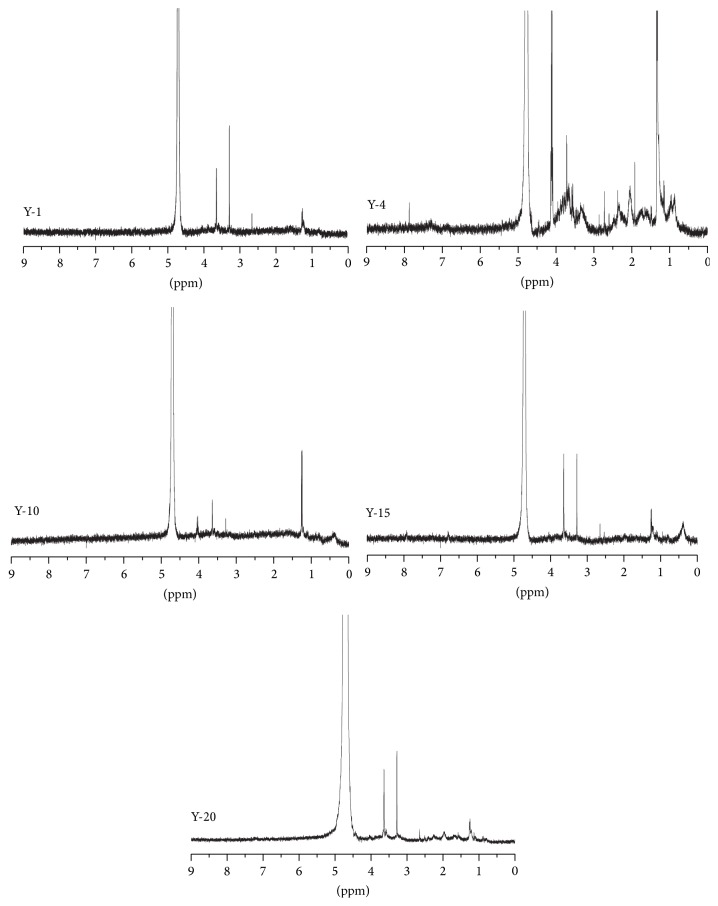
^1^H NMR spectra of DOM in the extracted samples of the five sampling sites.

**Figure 5:**
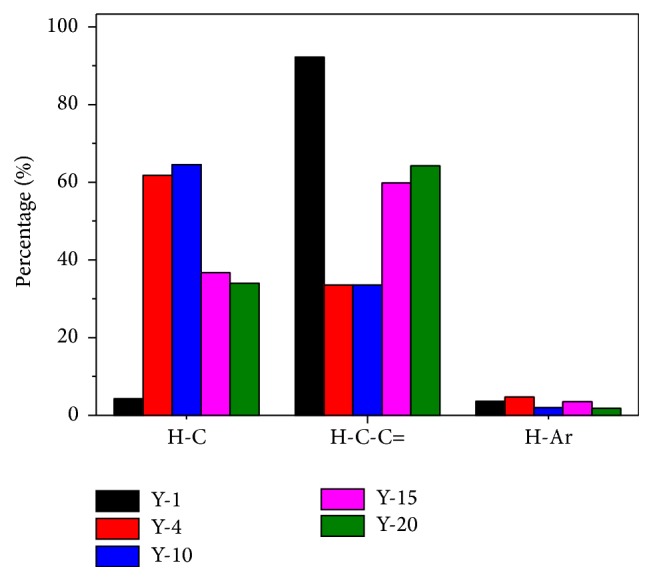
The relative abundance of each type of protons, estimated as the partial integrals of the spectra reported in Figure 4.

Proton NMR spectroscopy (^1^H NMR) was often utilized to characterize the composition of the DOM in soil samples, which can provide semiquantitative information on aromatic, aliphatic, and carboxylic groups [27, 44, 45]. Much different from UV-Visible spectroscopy and EEM, ^1^H NMR can give us the content and structural information via the corresponding integrated areas and the chemical shifts (*δ*_H_) [18, 19]. As illustrated in Figure 4, the ^1^H NMR results of the five sites exhibit some distinct peaks overlaying bands, implying the existence of complicated mixtures in the DOM. Despite the large variety of overlapping resonances, each ^1^H NMR spectrum was analyzed based on the chemical shift assignments following the method described in the previously reported literatures for soil DOM [27]. The integrated regions in the ^1^H NMR spectra were listed as follows: *δ*_H_ = 0.5–2.9 ppm (aliphatic protons, H-C); *δ*_H_ = 3.0–4.2 ppm (carbohydrates, H-C-C=); and *δ*_H_ = 6.0–8.0 ppm (aromatic protons) [27]. The spectra of five sites were depicted in Figure 5. The ^1^H NMR results of the five sites exhibited similar patterns as to functional group composition, suggesting the presence of more aliphatic and carbohydrates structures and less quantity of aromatic organic matters (Table 6).

The content of saturated aliphatic substances in site Y-1 (4.2%) was lower than other sites (68.1%, 64.5%, 36.7%, and 34.0% for Y-4, Y-10, Y-15, and Y-20, resp.), while the content of carbohydrates (92.2%) was higher than other sites (33.5%, 33.5%, 59.8%, and 64.2%, for Y-4, Y-10, Y-15, and Y-20, resp.). The content of aromatic structures in the five sites was 3.6%, 4.7%, 2%, 3.5%, and 1.8% for Y-1, Y-4, Y-10, Y-15, and Y-20, respectively. The results implied that the saturated aliphatic chains and carbohydrates were the main structures in afforestation forest land.

## 4. Conclusions

With this study, a preliminary soil quality evaluation were carried out based on soil texture and some chemical indicators like TOC, TN, TP, K^+^, MBC, and DOC. Particularly, the DOM in the soil samples with different afforestation time was further characterized via DOC, UV-Visible spectroscopy, EEM fluorescence spectroscopy, and ^1^H NMR spectroscopy. The results of EEM fluorescence spectroscopy demonstrated that UV humic-like substances were the major fluorophores components in the DOM of the soil samples. The DOM in the soil samples was mainly composed of aliphatic chains and aromatic components with carbonyl, carboxyl, and hydroxyl groups. DOM in soils plays a crucial role in soil physical, chemical, and biological processes, but little information is available on the formation and biodegradability of plant-derived DOM in afforestation forest soil. With the development of afforestation forest in Beijing, it is necessary to further investigate the DOM distribution and the corresponding influence on soil quality, plants growth, and ecosystem.

## Figures and Tables

**Table 1:** Locations and afforestation information of the samples.

Abbreviation	Tree species	Growing period	Coordinates
Y-1	Pagoda tree	1 yr	N40°24′59.6′′, E116°45′43.7′′
Y-4	Pagoda tree	4 yrs	N40°24′30.8′′, E116°45′00.0′′
Y-10	Populus tomentosa	10 yrs	N40°23′58.4′′, E116°43′41.6′′
Y-15	Populus tomentosa	15 yrs	N40°24′00.7′′, E116°43′37.1′′
Y-20	Shrub and locust	20 yrs	N40°20′52.2′′, E116°49′22.0′′

**Table 2:** Basic physical properties of the soil samples.

Sample point	Clay (%)	Silt (%)	Sand (%)	Bulk density (g·cm^−3^)
Y-1	0.21	7.15	91.23	1.57
Y-4	0.94	20.75	78.30	1.49
Y-10	0.82	23.24	75.94	1.45
Y-15	0.25	10.04	89.71	1.51
Y-20	1.14	24.97	71.89	1.51

**Table 3:** Basic chemical properties of the soil samples.

Sample point	pH	TN (g·kg^−1^)	TP (g·kg^−1^)	Available K (mg·kg^−1^)	TOC (g·kg^−1^)	MBC (mg·kg^−1^)	DOC (mg·kg^−1^)
Y-1	6.34	0.03	0.09	0.51	2.64	16.28	81.10
Y-4	6.33	0.05	0.19	0.30	2.77	42.09	68.45
Y-10	6.57	0.02	0.21	0.25	4.45	19.64	61.25
Y-15	6.65	0.02	0.48	0.21	4.52	59.45	56.08
Y-20	7.05	0.03	0.05	0.61	4.45	92.70	69.43

**Table 4:** Peaks, description, and excitation/emission maxima of fluorescent DOM.

Peaks	Description	Excitation max (nm)	Emission max (nm)
A	UV humic-like, less aromatic	<260	380–460
C	Visible humic-like, more aromatic	320–360	420–460
M	Marine-humic-like	290–310	370–410
B	Tyrosine-like substances	260	280
T	Protein-like tryptophan	250–300	305–355

**Table 5:** Results of UV-Visible spectroscopy and EEM fluorescence spectroscopy.

Sample point	SUVA_254_ (mg^−1^·m^−1^)	SR	FI	BIX	HIX
Y-1	5.39	0.64	1.54	0.56	14.7
Y-4	5.90	1.07	1.61	0.48	18.90
Y-10	4.83	0.92	1.46	0.57	2.17
Y-15	5.33	0.95	1.52	0.43	6.16
Y-20	4.39	0.77	1.51	0.67	3.66

**Table 6:** Results of the ^1^H NMR analyses.

Sample sites	% aliphatics (0.5–3.00 ppm)	% carbohydrates (3.00–4.2 ppm)	% aromatics (6.00–8.00 ppm)
Y-1	4.2	92.2	3.6
Y-4	61.8	33.5	4.7
Y-10	64.5	33.5	2
Y-15	36.7	59.8	3.5
Y-20	34	64.2	1.8
